# Does accreditation stimulate change? A study of the impact of the accreditation process on Canadian healthcare organizations

**DOI:** 10.1186/1748-5908-5-31

**Published:** 2010-04-26

**Authors:** Marie-Pascale Pomey, Louise Lemieux-Charles, François Champagne, Doug Angus, Abdo Shabah, André-Pierre Contandriopoulos

**Affiliations:** 1 Department of Health Administration, GRIS, Faculty of Medicine, University of Montreal, CP 6128, Succ. Centre Ville, Montreal, Québec, Canada H3C 3J7; 2 Department of Health Policy, Management and Evaluation, University of Toronto, Canada; 3 Telfer School of Management, University of Ottawa, 55 Laurier Avenue East., Ottawa, ON, K1N 6N5, Canada; 4 Direction de la santé publique de Montréal, 1301 Sherbrooke Est, Montréal (Québec), H2L 1M3

## Abstract

**Background:**

One way to improve quality and safety in healthcare organizations (HCOs) is through accreditation. Accreditation is a rigorous external evaluation process that comprises self-assessment against a given set of standards, an on-site survey followed by a report with or without recommendations, and the award or refusal of accreditation status. This study evaluates how the accreditation process helps introduce organizational changes that enhance the quality and safety of care.

**Methods:**

We used an embedded multiple case study design to explore organizational characteristics and identify changes linked to the accreditation process. We employed a theoretical framework to analyze various elements and for each case, we interviewed top managers, conducted focus groups with staff directly involved in the accreditation process, and analyzed self-assessment reports, accreditation reports and other case-related documents.

**Results:**

The context in which accreditation took place, including the organizational context, influenced the type of change dynamics that occurred in HCOs. Furthermore, while accreditation itself was not necessarily the element that initiated change, the accreditation process was a highly effective tool for (i) accelerating integration and stimulating a spirit of cooperation in newly merged HCOs; (ii) helping to introduce continuous quality improvement programs to newly accredited or not-yet-accredited organizations; (iii) creating new leadership for quality improvement initiatives; (iv) increasing social capital by giving staff the opportunity to develop relationships; and (v) fostering links between HCOs and other stakeholders. The study also found that HCOs' motivation to introduce accreditation-related changes dwindled over time.

**Conclusions:**

We conclude that the accreditation process is an effective leitmotiv for the introduction of change but is nonetheless subject to a learning cycle and a learning curve. Institutions invest greatly to conform to the first accreditation visit and reap the greatest benefits in the next three accreditation cycles (3 to 10 years after initial accreditation). After 10 years, however, institutions begin to find accreditation less challenging. To maximize the benefits of the accreditation process, HCOs and accrediting bodies must seek ways to take full advantage of each stage of the accreditation process over time.

## Introduction

Today's healthcare organizations (HCOs) struggle with paradoxes of all kinds. They must reconcile multiple goals, such as teaching students and caring for patients, with different modi operandi (managerial, professional, technocratic, and others) [1,2]. They must give doctors the freedom to exercise their clinical judgment while promoting the standardization of practices [3]. They must act autonomously, yet in coordination with community players, and they must both meet expectations and innovate. In addition, they are under increasing pressure to improve performance, as a number of recent publications have reported serious shortcomings in the quality and safety of services and care [4-8].

One of the ways in which countries around the world have sought to improve performance is through accreditation [9-12]. A literature review of the impacts of accreditation on HCOs suggests that more research is necessary to determine whether accreditation truly improves healthcare services delivery and health outcomes [13]. This is certainly the case in Canada, where even though accreditation through the United States' Joint Commission of Healthcare Organizations dates from the beginning of the twentieth century, little is known about the real impacts of the accreditation process on Canadian HCOs [14-19]. Still, recent government-commissioned reports that recommend making accreditation obligatory for all HCOs demonstrate the prevalence of Canadians' assumption that accreditation is a guarantee of a high level of quality and safety of care [6,7].

Given this background, our study aimed to clarify the impacts of accreditation in Canada by asking the following question: what kind of organizational changes does the accreditation process introduce within HCOs?

To answer this question, we analyzed changes that occurred during a recent accreditation cycle in five Canadian HCOs. The lack of result indicators during the period of study prevented us from assessing the impact of accreditation on patient outcomes. Rather, we identified the principal organizational changes that occurred during the accreditation cycle.

### Overview of accreditation in Canada

In Canada, questions of the quality of care fall mainly to the provinces, where they have principally been treated as a professional concern, with the provincial college of each medical specialty regularly monitoring its members. In addition, Accreditation Canada (formerly the Canadian Council on Health Services Accreditation--CCHSA) helps guarantee uniformity throughout the Canadian system. A member of the International Society for Quality in Health Care [20], Accreditation Canada is a national, non-profit, independent organization that was created in 1958 to help guarantee that healthcare organizations across Canada furnish services of acceptable quality. Accreditation Canada follows international accreditation rules regarding HCOs' self-assessment against a given set of standards, an on-site survey followed by a report with or without recommendations, and the award or refusal of accreditation status. The standards are determined by professional consensus.

The understanding between the accrediting body and the HCO is that the information in the accreditation visit report remain strictly confidential. However, a list of accredited establishments is published on the Accreditation Canada website. In Canada, accreditation surveyors must adhere to their role as evaluators and quality advisors, not whistle-blowers, although those who notice significant problems tend to notify the authorities. Finally, even though accreditation in Canada is voluntary (except for First Nations' facilities, university-affiliated hospitals, and since 2005, institutions in the province of Quebec [21]), 99% of Canada's short-term stay institutions, 85% of its mental health establishments and 80% of its long-term care institutions participate in accreditation [22].

### Theoretical framework

**Figure 1:**
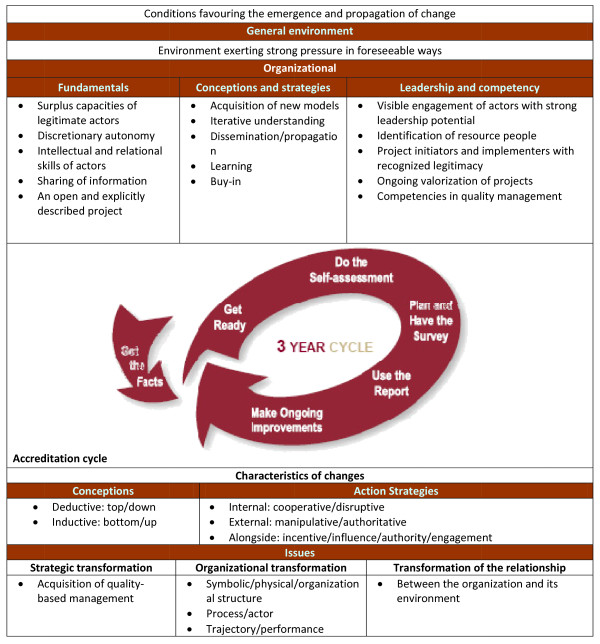
**Conditions and characteristics of change **[24].

To study the changes that took place in five Canadian HCOs as a result of the accreditation process, we employed a theoretical framework that had previously been used to analyze organizational changes in a French HCO during the self-assessment phase of accreditation [23,24]. Based on the literature on the theory of change, this framework inventories changes that take place as a result of the accreditation process and explores the impact of internal and external conditions (Figure 1). The features of the changes are studied in terms of their characteristics (conceptual approach and action strategies) and their issues (strategic transformation, organizational transformation and transformation of the relationship). Insofar as internal and external conditions are concerned, four factors are seen to promote change: (1) an environment that exercises external pressure and allows a project to go forward; (2) the existence of certain basic factors; (3) a realistic conceptual approach and specific implementation strategies; and (4) appropriate skills and leadership.

While our study is exhaustive in its listing of the changes that took place in the institutions studied, the number of case studies and the number of changes obliged us to limit our discussion to the most significant ways in which organizational changes related to contextual conditions.

### Study design and methods

Between 2003 and 2005, we conducted an in-depth retrospective case study [25] of five HCOs with different accreditation statuses. Rather than aim for the best possible internal and external validity [26,27], we chose to assess a small number of cases in detail [28,29], conducting a multi-case study with multiple levels of analysis [26,29].

#### Case selection

The literature suggests that context often has an important influence on organizational change [30]. For that reason, we selected cases that represented a variety of accreditation situations in Canada but still followed the same accreditation program: Achieving Improved Measurement [31]. This meant that all cases possessed the same comprehensive accreditation report. We used three selection criteria simultaneously. The criteria were chosen by the research team for their particular importance to the Canadian context. The first criterion was geographical location. We wished cases to represent Canada's four general cultural zones: the Western and prairie provinces (British Columbia, Alberta, Saskatchewan and Manitoba), Ontario (Canada's most populous province), Quebec (Canada's only French-speaking province), and the Atlantic provinces (Nova Scotia, New Brunswick, Newfoundland and Labrador, and Prince Edward Island). The second criterion related to HCOs' organizational structure. Substantial structural reforms have taken place in Canada over the past 20 years, giving rise to three kinds of establishments, largely organized by geographical region: 1) regional health authorities (RHAs) in the Western and Atlantic provinces, 2) merged academic HCOs in Ontario, and 3) hospitals in Ontario and Quebec. The third and last criterion regarded accreditation status, namely, the length of time the HCO had been engaged in accreditation. A Canadian study [17] showed that changes within HCOs differed according to the number of years the HCOs had spent participating in accreditation. In other words, changes varied according to whether an HCO was in its first accreditation cycle, had already experienced several cycles, or had participated in accreditation for over 10 years. To reconcile these criteria, we asked Accreditation Canada for a list of HCOs that participated in accreditation with the HCOs' location, their type of organization, and the number of years they had been involved in the accreditation process. With this information, we chose five establishments that represented the diversity of Canada's HCOs at the time of selection. This allowed us to follow Creswell's recommendations for qualitative research and study several cases in depth in order to maximize lessons learned.

The five cases retained were as follows: a RHA in Alberta that had participated in accreditation for the first time (Case 1); an urban hospital in Ontario that had participated in accreditation for many years (Case 2); an academic center in Ontario that had recently merged into a newly accredited HCO, the constituent institutions of which had all been previously accredited (Case 3); a semi-rural hospital in Quebec that had been accredited for many years (Case 4); and a RHA in New Brunswick that was newly accredited, the pre-merger institutions of which had all been accredited in the past (Case 5). Table 1 summarizes the characteristics of each case.

**Table 1:** Profiles of the cases

General characteristics	Case 1: Rural regional health authority	Case 2: University healthcare center	Case 3: General hospital	Case 4: Local hospital	Case 5: Urban regional health authority
**Province**	Alberta	Ontario	Ontario	Quebec	New Brunswick

**Location**	Sub-rural	Urban	Urban	Rural	Urban

**Population served**	300,000	1,500,000	400,000	135,000	86,000

**Number of employees**	8,000 staff and 350 physicians	10,600 staff and 1125 physicians	2,400 staff and 400 physicians	1037 staff and 102 physicians	2,600 staff and 340 physicians

**Number of sites and beds**	35 sites and 1300 beds	3 sites and 1099 beds	2 sites and 500 beds	1 site and 303 beds	8 sites and 425 beds in 2 hospitals

**Date of accreditation visit studied; accreditation status awarded**	2002; accreditation with report (3 key recommendations and 3 recommendations)	2004; accreditation (9 recommendations and 9 good practices)	2003; accreditation with report (20 key recommendations, 18 recommendations and 1 good practice)	2003; accreditation with report (9 key recommendations and 3 recommendations)	2002; accreditation with report (3 key recommendations and 2 good practices)

**Length of participation in the accreditation process**	Since 2002	Since 2000 for the new entity	Since 1951	Since the 1980s	Since 1998 for the new entity

**Number of accreditation teams**	15 clinical teams 4 support teams	17 clinical teams 4 support teams	8 clinical teams 4 support teams	8 clinical teams 4 support teams	8 clinical teams 4 support teams

**Research site visit dates**	November 1 and 2, 2004	June 16 and 17, 2004	December 5 and 6, 2004	June 21 and 22, 2004	June 1 and 2, 2004

**Type of accreditation**	Non compulsory	Compulsory	Compulsory	Non compulsory	Non compulsory

#### Data collection methods

The use of multiple data sources is helpful in generating complex theories and strengthening empirical grounding [32]. Our use of multiple sources allowed us to address a wide range of issues and obtain a nuanced understanding of the context of events that affect the relationship between accreditation and changes in quality. Accordingly, we collected retrospective data via document analysis, 25 interviews and 10 focus groups. Insofar as documents were concerned, we accessed both the HCOs' self-assessment reports and their accreditation reports. For interviews, we talked to chief executive officers (CEOs), quality directors/vice-presidents, human resources directors/vice-presidents, medical directors/vice-presidents and nurse directors/vice-presidents with a view to discerning top management's perception of the impact of the accreditation process. We conducted between five and seven interviews at each site and for each interview, we used a semi-structured questionnaire composed of four sections adapted from the study in France and previously tested in two Canadian HCOs (one French-speaking and one English-speaking). Our focus groups were designed to obtain the perceptions of staff. Accordingly we conducted two focus groups at each site, one with a sample of employees who had been involved in the clinical self-assessment team (between 8 and 10 employees per site) and another with a sample of employees who had been involved in the support self-assessment team (i.e., employees from the Leadership and Partnership Team, the Environment Team, the Information Management Team and the Human Resources Team; between five and eight employees per site). In the focus groups, we again used a semi-structured questionnaire with the same four sections, also tested in English and French. Each interview or focus group lasted one to two hours. All were taped and transcribed for analysis with N-Vivo. The composition of each focus group was determined by the site's quality director in concert with the primary author and was made up of representatives from departments across the HCO. In total, 67 participants were involved in this study: 25 in interviews and 42 in focus groups.

#### Data analysis

For each case, the interviews and the focus groups were transcribed and processed using N-Vivo software (QSR International). The documents were also analyzed using N-Vivo. All data were examined in light of our theoretical framework. To cross-compare cases, we used techniques for data reduction and presentation similar to those suggested by Miles and Huberman [33,34]. Research team members collectively analyzed and interpreted the results using deductive methods related to our theoretical framework. Our research team was staffed by professionals from a variety of backgrounds, namely, economics, public health, sociology, management, medicine, and nursing. In order to validate our analysis, we forwarded a preliminary research report to each quality director for comment [35-39]. Our interpretation of the entire set of data integrates these directors' feedback and their validation of our results.

## Results

In this section, we present the conditions of change and the organizational changes that occurred during the accreditation cycle studied, for each case. A summary of the conditions favoring organizational change are presented in Table 2.

**Table 2:** Conditions favouring organisational changes

Determinants	Case 1	Case 2	Case 3	Case 4	Case 5
General environment	Serious financial problems and major financial cuts.	New provincial accountability agreement.	Presence of the Foundation of Leadership and its Thousand and One Leaders Program.	Financial pressure.	Absence of a faculty of medicine Few opportunities for external recognition.

Fundamentals	Merger into a single region. Quality of care and client-centering recognized as important values. Teamwork and creativity encouraged	Merger of three hospitals. Increase in cognitive capacities by hiring new staff with higher qualifications and experience. Autonomy encouraged.	Placement under the guardianship of a supervisor in 2001 and again in 2002. New board committee structure and a new set of board policies. A new CEO appointed in 2003. High turnover of personnel.	Increasing services offered to meet to the needs of the local population Recruitment campaign to hire 50 physicians. Good relationships with the ministry of health.	Merger into a RHA Appointment of a new board. Focus on patient care.

Strategies	Creation of forums where leadership seeks staff input; numerous newsletters; online chats; investigative teams frequently created to inform quick decisions.	Surveys, regular visits from vice-presidents, regular meetings of professional teams. Communication plan for the entire hospital for every decisions taken by the board of directors	Managers meet monthly with clinical and support assistants; multidisciplinary unit councils make decisions for major initiatives Professionals are consulted on all matters	Horizontal exchanges of ideas and horizontal learning and dissemination of information.	Training courses, including incident reporting system; audits; patient surveys; benchmarking.

Leadership and Competencies	Strong leadership by experienced management at all levels CEO's involvement in QI. Creation of a quality department and quality teams for the accreditation process.	High level of leadership dissemination. CEO's personally involved in QI	Member of the Foundation of Leadership and its Thousand and One Leaders Program. Strong legitimacy of the quality director	Strong leadership by the CEO. Focus on outcomes and not processes -	Leadership for QI encouraged at all levels Director of QI and Risk Manager seen as leaders.

Conceptualization /Philosophy	Developed a confident and accountable method of decision-making.	Seemed to have the ability to critique itself.	Seemed keen to accept new model of thinking.	Felt the duty to meet public expectations.	Presented a certain lack of self-worth

### Case 1

A newly created RHA made up of the merger of several HCOs, none of which had previous experience with the accreditation process.

#### Conditions for the implementation of change

Alberta in the early 1990s was experiencing serious financial problems that caused cuts to healthcare services. These cuts mandated a more integrated healthcare system with lower spending and more stable funding. In 1994, Alberta's Regional Health Authorities Act established 17 autonomous health regions. In 1998, Alberta's per capita health spending dropped to the lowest in Canada. In 2003, the 17 health regions were reduced to nine.

The consensus from study participants was that leadership was strong and concerned not only the CEO but management at all levels. Both medical and informal leadership were recognized. Changes were sometime unexpected and were sometimes economically or politically driven, but even as the organization expanded, its workers and their knowledge of history remained, giving staff stability and a sense of continuity. Because of frequent changes and stable leadership, this RHA had developed a confident and accountable decision-making approach.

#### Changes during the accreditation cycle

It was clear the changes during the self-assessment phase were substantial; indeed, the most important changes implemented during the accreditation cycle had been identified during self-assessment. Preparations for accreditation were mostly conducted by the new quality control entity, and nurse managers were mainly in charge of organizing the process. The RHA mainly used accreditation to integrate the pre-existing entities into the new entity. It instituted a Quality Department and Quality Improvement Teams specifically for the accreditation process, and the self-assessment phase created the opportunity for individuals from different sites to meet, begin to overcome their differences and start seeing themselves as part of one new organization. The RHA was a large organization composed of a number of facilities spread over a wide geographical area. The accreditation process also proved to be a means for the RHA to involve community members in decision-making and determination of the organization's orientation. Before the accreditation visit and the report, the RHA had already worked to remedy some of its problems:

"There were major issues that my team identified. Some of them sort of overlapped into each other as well, and one of them was related to fire drills across the region. There were no documented standards according to which [the drills] should occur, and there was no documentation to identify what to do in case of fire. So actually once it was identified, there had been, before the surveyors even came, there was some work being done on trying to correct that problem." (Case 1 - Clinical Focus Group)

Respondents considered that accreditation's highlighting of problem areas helped the institution set priorities and accelerate procedures to implement change because of the pre-determined structure of the accreditation process, which required participants to answer to the accrediting body regarding matters where change was expected. In addition, the Quality Steering Committee asked each self-assessment team to name its top three priorities and identify eight to ten regional priority areas for the entire organization to start working on before the surveyors arrived and/or the final report was issued.

Many of the resulting changes took place at the public health level (the interconnection of immunization registries and community mapping) and at the clinical level (new space and equipment in the nursery unit, new evidence-based practices in maternal child and palliative care, and new ambulatory and emergency services planning).

"So for the continuing care team, following the accreditation report, on one hand the best practices team took all the suggestions... to improve and develop practices, and on the other hand, it set priorities and incorporated them into our operational plan wherever they needed to be" (Case 1 - Support Focus Group).

Several improvements also occurred at the management level: a new information management strategy was created, a new performance appraisal process was implemented, and the positions of director of human resources and education officer were merged. At the regional level, a security and incidents committee, a research committee and an ethics committee were set up.

### Case 2

An academic healthcare facility in Ontario that had recently merged into a new HCO and was experiencing its first accreditation cycle. All three pre-merger institutions had been accredited in the past.

#### Conditions for the implementation of change

The greatest environmental pressure exerted on this hospital was the 1998 merger that created it subsequent to a decision by the Ontario Health Services Restructuring Commission. A provincially legislated accountability agreement was also increasing financial pressure: in the words of one interviewee, the hospital had already been under an 8-year "fiscal siege". Regarding organizational conditions, the hospital encouraged a high degree of autonomy, which facilitated the implementation of change. In addition, Board of Directors meetings were open to all staff members, who were welcome to participate in Board decisions. The CEO also held regular open forums where employees had the opportunity to learn about management decisions and could express their concerns. Professional development was encouraged via professional teams that met regularly and the hospital had a high level of leadership diffusion, meaning that all levels of staff, from nurses to senior management, were involved with and responsible for creating quality initiatives. The hospital tried to hire physicians with leadership and administration skills, and these personnel, along with the leadership of key senior managers, was helping the institution become recognized as a leader in some areas, especially quality and patient safety, both within the community and nationally. Finally, stakeholders were encouraged to participate in the institution's functioning.

#### Changes during the accreditation cycle

While this was the new, integrated HCO's first accreditation process, all three pre-merger institutions had been accredited for over 5 years. The accreditation process took place just a few months after the merger and was conducted by nurse managers who were also in charge of quality improvement. Doctors' participation varied by self-assessment group, but overall, doctors did not much participate. Despite a history of competition, the three sites were obliged to work together during the accreditation process. At the beginning of the self-assessment phase, staff seated around the table had divided into three groups, each of which spoke to the moderator but not to the other groups. By the end of the self-assessment phase, staff from different sites sat in mixed groups around the table. They also exchanged protocols, discussed means of implementing common working procedures, and collaborated on better integrating the patient pathway within the organization. In this way, even though accreditation was not linked to the merger per se, the CEO felt that it served to accelerate the merging process.

"In the process of merging, accreditation showed no impact on the merger decision itself: this was a strong external process solely directed by outside forces. But it showed great impact as a framework to speed and share a totally new culture." (Case 2 - CEO's Interview)

No changes took place during the site visit. After the visit, most changes resulted from the accreditation report. Three changes affected group practices: social work hours in the intensive care unit were increased, medical quality improvement and risk indicators and activities were incorporated into the institution's quality program, and a pain management tool was developed and implemented. Additional changes involving the entire organization concerned new, improved reporting mechanisms on safety, quality, and risk, including adverse events; the resolution of space and equipment issues in ambulatory care; and the implementation of an ethics committee. The accreditation report had mentioned the need to centralize rehabilitation services and to collect information on population health determinants such as obesity, smoking, and poverty. As a result, the HCO solicited the help of the provincial government in securing capital for new ambulatory services oriented toward rehabilitation, risk prevention and new emergency services. The accreditation report also underlined the importance of maintaining good communication with the community, especially in times of change and uncertainty, in order to establish good partnerships. Our respondents also raised a negative aspect of accreditation. During the accreditation process, the palliative care assessment team had been highly commended as one of the organization's strengths. After the accreditation report brought other issues to the attention of top managers, however, this team lost much of its support.

### Case 3

An Ontario hospital that had been accredited for many years.

#### Conditions for the implementation of change

This hospital had a tumultuous history, having been placed under the guardianship of a provincial supervisor in 2001 and again in 2002. The supervisor developed key governance documents, a new Board of Directors committee structure with new terms of reference, and a completely new set of Board policies and corporate by-laws, all designed to re-establish good governance. As a result, the organization adopted various decision-making bodies such as unit councils and a Performance Improvement Committee. Professionals were consulted on matters relative to their field of expertise but not on budget-related issues, which fell to health service directors. The organization also joined the Foundation of Leadership and its Thousand and One Leaders Program. Under this initiative, training programs in leadership skills took place four times a year. A key component of these programs was the group project developed by program participants. Working in leaderless groups, participants presented their project on "Capstone Day," a day of presentations at the end of term. All senior leadership attended Capstone Day and a graduation ceremony followed the presentations. In this way, the organization distinguished those with the skills to be leaders and encouraged others to follow the program likewise. The quality director had strong legitimacy within the organization and a sound knowledge of quality issues.

#### Changes during the accreditation cycle

For this institution, accreditation's self-assessment phase no longer represented a challenge. The institution was obliged to be involved in the accreditation process because it was a university centre. The organization of the accreditation process was assigned to the quality control entity, which was staffed exclusively by nursing staff. Doctors' participation was more anecdotal than consistent and depended on the personal interest of each doctor. No changes occurred during the site visit. After the visit, and despite the fact that the accreditation report made recommendations, respondents did not consider accreditation to be a driver of change but rather a recurrent introspective exercise that instigated or enhanced other quality measures and identified areas where quality ought to be improved. This organization was principally oriented towards Canada's National Quality Institute and its norms for organizational quality and wellness. These norms were consistent with the goals of the institution and its CEO, namely, strengthening the organization's leadership and the quality of life of its staff.

Among measures undertaken by the HCO pursuant to the accreditation process were several initiatives designed to encourage leadership. These included training programs, a board-level balanced scorecard, and participation in the National Quality Institute program. Staff turnover rates in certain services and occupational categories had been high and after the report was released, the HCO put new emphasis on staff retention strategies such as an orientation program, conferences, and partnership councils. Another important change was the adoption of an accountability framework. This framework was part of the accreditation report's key recommendations and helped the organization discuss the kinds of outcome indicators that would help it make decisions at different levels.

### Case 4

A Quebec hospital that had been accredited for many years.

#### Conditions for the implementation of change

The chief executive of this HCO demonstrated exceptionally strong leadership and marked entrepreneurial qualities, for example with regard to fundraising. Under his leadership, this hospital broadened its range of services and recruited 50 new physicians. In 2003, the institution made quality improvement functions into regular institutional activities and named a staff member to head matters related to quality, risks, complaints and the prevention of nosocomial infections. It also created an ethical committee, a multilingual committee, a committee on pain management and a committee on quality. The fact that the hospital had a single location made it easy for staff members to know each other. As was fitting for the hospital's size, strategies for exchanging ideas, learning, and sharing information consisted mainly of oral communication. The institution valued the qualities of each actor and the organizational culture was considered to be open to change. Managers and professionals were young and dynamic. They communicated extensively in order to implement change efficiently and quickly. Members of the Board of Directors were also very active: they represented a cross-section of the region's economic make-up and the CEO listened to them carefully. The hospital had deep roots in the local population and staff felt it incumbent on them to meet public expectations.

#### Changes during the accreditation cycle

For the CEO, the accreditation process was a good way to prioritize the organization's objectives and to discuss with financial authorities how to implement the recommendations of the accrediting body. Although preparation for accreditation had been assigned to nurse managers, doctors participated actively as well after the director of professional services succeeded in motivating her colleagues to take part in various working groups. During the self-assessment phase of accreditation, the HCO hired a consultant to help organize the accreditation process around the hospital's quality improvement program. Starting from the hospital's most recent accreditation report, staff created a template to monitor changes that were required and changes that were implemented. This exercise allowed them to link accreditation standards to changes actually made. Nothing notable occurred during the site visit, and the organization was accredited with a report that included key recommendations. All recommendations corresponded to problems that the organization had pointed out to the surveyors during the site visit. The CEO was grateful for the recommendations because they gave him a tool with which he could emphasize the institution's needs to the provincial ministry of health. By far the greatest impact of the accreditation process in this organization was the creation of an organizational structure dedicated to improving quality. This structure, temporary at first, took the form of committees composed of the representatives of various departments and followed the recommendations of Accreditation Canada. After accreditation in 2003, the CEO went a step further and integrated Accreditation Canada's quality objectives within the organization's mission.

"Were it not for Accreditation Canada, I am sure that we would not have adopted a specific structure for quality. We would have simply integrated quality within everyone's individual responsibilities, and as we all know, when you integrate, you minimize." (Case 4 - Clinical Focus Group)

Not only did the accreditation recommendations cause management to adjust and modify many practices, staff also used them to convince management and the Board of Directors to adopt particular measures such as the establishment of an ethics committee, a multilingual committee, a pain management committee and a quality improvement committee.

### Case 5

A newly accredited RHA in New Brunswick, the pre-merger institutions of which had been accredited previously.

#### Conditions for the implementation of change

In April 2002, this corporate institution became a RHA only 6 months prior to its scheduled accreditation survey. The change involved the appointment of a new Board of Directors. Chronic financial constraints in health care throughout New Brunswick had put pressure on the healthcare system and influenced the direction of change within the organization. For two years in a row (2004 and 2005), MacLean's magazine named this RHA one of Canada's 100 top employers, testimony to its excellent management of human resources. The absence of a provincial faculty of medicine made it difficult for the organization to recruit physicians and highly specialized staff. The RHA gave staff learning opportunities by providing training courses, including leadership training; by having staff shadow others when taking over a position; and by encouraging staff to participate in quality improvement team meetings and/or monthly program meetings. The Board also sought to develop its relationships with external stakeholders by presenting its services in the community. To encourage physicians to participate in decision-making, one full-time physician employed as the medical director of a program spent one day a week with the administrative program director. The former CEO, an Accreditation Canada surveyor, implemented a quality control and improvement program. The director of quality improvement and the risk manager were both mentioned by several respondents as leaders in their field and very visible in their organization. Several interviewees suggested that the RHA presented a lack of self-worth that was partially attributed to its isolation in a maritime province.

#### Changes during the accreditation cycle

Preparing for accreditation was assigned to the institution's research department, not to nursing staff. Doctors participated significantly at the management level but rarely in self-assessment activities. The main institution that made up this newly created RHA had participated in the accreditation process since 1998 but the accreditation cycle under study was the RHA's first since the merger. Working together in accreditation teams helped individuals from different sites learn about practices at other locations, share ideas and discuss their respective processes. Prior to the accreditation visit, this RHA had experienced problems with physicians failing to sign patient files. During the surveyors' visit, the CEO and the institution's medical director urged physicians to respond to accreditation requirements: "You cannot work until your charts are up to date and signed. Otherwise, your privileges are gone" (Case 5 - Accreditation coordinator). Immediately, a policy on the matter was developed with the goal that the situation be corrected before publication of the final report. As the quality director mentioned, "Basically they had been told for many years to sign their charts, which later on was corrected quickly. I think that's the value of accreditation." The status awarded to the RHA was accreditation with a report. The report included key recommendations and named two good practices. Respondents reported that staff viewed accreditation as a morale booster and a welcome opportunity to be compared to other Canadian organizations. Acting upon the recommendations of the hospital's accreditation report, the RHA created an ethics committee headed by a full-time ethicist. The accreditation report had also noted the need to improve processes related to patients' health records, including progress notes, and recommended that the RHA implement a coordinated corporate quality improvement structure to ensure the integration of continuous quality improvement throughout the organization. Acting upon the report's recommendations, the RHA began to implement a new quality improvement framework that included a standardized approach to quality improvement.

"So a form was developed to document pain management. Probably, we recognized that we knew that we needed to do that, but with accreditation it was a recommendation for improved programming so that has been done, and we've been using it." (Case 5 - Support Focus Group)

"One of the things that came out of accreditation was the ethics committee, and the interesting reaction was that we didn't hear of any action about it. A group of clinical instructors got together, and reviewed some of the things that were going on in the building, issues that we might identify, and brought it to the powers that be." (Case 5 - Clinical Focus Group)

## Discussion and recommendations

This study is the first of its kind in Canada to document the impact of the accreditation process on HCOs in terms of organizational changes. In Canada, where accreditation has taken place for almost a century, it is impossible to realize a quasi-experimental research design as has been done in Australia [40] or in South Africa [41]. We tried to compensate by ensuring the representativity of our cases and by having respondents discuss which of the organizational changes observed could be attributed to the accreditation process. Presentation of our results to professionals involved in accreditation at different levels of Canada's healthcare system allowed us to validate our findings. The congruence between our model of analysis and observations collected previously from various sources of data supports us in asserting the validity of this study.

This study reveals several findings that support the findings from other research. First, it shows that the ways that institutions use the accreditation process depends on the context in which accreditation takes place. For one HCO, for example (Case 5), accreditation was a means to compare its performance to the performance of other HCOs and to break its geographical isolation. This was also the experience of an institution in France, which feared that its provincial location excluded it from exercising its functions at the same level of quality as institutions in large urban centers [23]. For Case 5, accreditation was a means to confirm that what it did locally was comparable to what took place elsewhere. For another HCO (Case 3), accreditation was seen as an obligation: the institution's main goal was to obtain accreditation status. Case 4, in contrast, saw accreditation as a tool for soliciting the financial support of funding organizations in order to implement recommendations for improvement [42]. And finally, for the three HCOs that had undergone mergers (Cases 1, 2 and 5), accreditation was used as a management tool to cause the various sites of the newly merged entity to adhere to a new institutional identity and integrate common clinical practices, for example a collecting monitoring protocol. The self-assessment groups acted as forums for meditation and interpersonal exchanges that eventually allowed a new, common institutional culture to emerge, in accordance with the findings of McNulty and Ferlie (2002) [43] and in confirmation of Fulop's observation that [44] "perceived differences in cultures seem to form a barrier to bringing organizations together." Still, these results should be validated in other contexts.

Second, the study showed that the pressures caused by the difficult economic environment of the end of the 1990s and the early 2000s caused HCOs to cut back or eliminate their quality programs, even when those programs had been part of the accreditation process for some time. This phenomenon had been observed in Quebec [14,15] but had not been studied in the other provinces. Subsequent pressure caused by publicity around serious medical accidents in Canadian HCOs [45] gave renewed legitimacy to the institutional quality structures and programs that the accreditation manual had advocated all along.

The third finding of this study concerns the paradox of success. In Case 2, the accreditation process recognized the accomplishments of the palliative care assessment team, following which the team lost momentum as a result of its funding being redirected to more problematic areas. This showcases the fact that accreditation should not only be used to find problems but also to validate and recognize success. Without this mandate, the accreditation process will undermine the very goals it hopes to reach.

Fourth, the study showed that different phases of the accreditation process caused different kinds of changes to occur. The self-assessment phase lent itself well to self-reflection and the identification of problem areas [23]. This was the phase that built consensus for the changes that the institution saw as most important and most legitimate. The accreditation visit phase resulted in relatively few changes, except when accreditors pointed out deviations to regulations [46] or when security was at stake [18,46]. Finally, in the last phase of accreditation, namely the period that follows the reception of the accreditation report, the HCO essentially responded to the report's recommendations in order to achieve accredited status.

Other less novel findings of this study corroborate or nuance the findings of other studies in related areas. One such area concerns doctors' participation in the accreditation process. In most cases, doctors' participation was characterized as weak (Cases 1, 2 and 5) or inexistent (Case 3) and directors of quality departments and nurse managers were those most involved in accreditation [14,23,40,45,47,48]. When doctors did participate, only a few individuals personally interested in quality processes and risk management actually took part [47,49]. Even directors of professional services showed little interest in the benefits of the accreditation process, seeing it as a procedure principally relevant to managers and nurses. Only in Case 4, a small institution where directors knew each other personally, did physicians participate more actively, cognizant of the importance of accreditation to the institution's funding. This phenomenon showcases a real problem with the way that the accreditation process takes place within HCOs [49]. In response, Accreditation Canada's new manual, Qmentum, includes questionnaires for all actors, and doctors are strongly encouraged to participate. Accreditation Canada has also reoriented its manual towards patient security, knowing that doctors are particularly concerned by the threat of malpractice suits [45,50-52].

Pomey et al's study in France [23] showed that the self-assessment phase is opportune for the creation of capital social, defined by Bourdieu [53] as the ability to create a durable network of social relations or to develop membership in a stable group that the individual can mobilize as part of his action strategies. Our study demonstrates this phenomenon in the context of mergers, where three HCOs used self-assessments to build relationships with individuals with whom they had previously been in conflict or with whom they had not been in contact because of the size of the territory and the number of sites involved. In these cases, accreditation quickly created social links [54].

The study also showed that accreditation causes certain practices to be modified. Accreditation has, for example, occasioned the more structured and systematic collection of quality and security-related data [11,55]. Canadian studies by Lemieux-Charles et al [17,56] have shown that this data had been seldom collected in the past. The fact that AIM standards include the implementation of indicators, even though specifics of those indicators are not given, has already caused institutions to change their practices and shows that accreditation results in the creation of various committees. This phenomenon has been observed in other studies as well [14,23,40,57].

This study also shows that the number of years that an HCO has participated in accreditation can affect the extent of the changes that take place. It seems that initially, institutions invest greatly in order to learn how to conform to the first accreditation visit and reap the most benefits possible from accreditors' diagnosis and the ensuing changes (Cases 1, 2 and 5). After 10 years, it would appear that institutions no longer find accreditation challenging, even if they are given recommendations (Case 2) and are looking for other external procedure with which to challenge themselves. This finding suggests that further research study the learning curve associated with accreditation [58-60].

At the external level, the accreditation process served to involve patients and families in quality management (Case 2). The process was an opportunity to enhance current relationships, bring new partners together and create common ground and standards (Cases 1, 2 and 5) [61].

To conclude, we use the findings detailed above to make several recommendations to policy makers, accrediting bodies, managers of healthcare organizations and researchers.

At the policy-making level, these initial results regarding the impact of accreditation on mergers suggest that accreditation should be seen as a tool for the structural and clinical integration of the newly merged entity.

Accrediting bodies should look into putting the entire accreditation process to use and finding new ways to sustain motivation in HCOs after the 10-year point. It is important that entities in this position review the accreditation process on an ongoing basis in order that it remain an impetus for HCOs to continue to improve quality [62]. It is also important that accreditation bodies take physicians' disengagement from the accreditation process seriously and devise means to increase doctors' involvement. We have mentioned a few initiatives on the part of Accreditation Canada but further measures should be explored, for example by ISQUA. Accreditation bodies should also make better use of the three phases of accreditation. Some organizations [35] have considered leaving self-assessment to HCOS and concentrating accrediting activities on the accreditation visit and the implementation of the recommendations of the accreditation report. Finally, it would be important for accrediting bodies to not only concentrate on problem areas but also recognize and encourage successful initiatives and teams. One Accreditation Canada initiative in this sense is to share information about good practices among establishments.

At the HCO level, there is always the risk of accreditation becoming the purview of a few isolated specialists and/or being more and more confined to nursing staff.

With respect to research, finally, this study, like that of Braithwaite and colleagues [63], suggests the importance of better understanding how accreditation can help mergers, how the learning curve functions with regard to the number of years for which HCOs have been involved in accreditation, and what can be done to bring more doctors on board.

## Declaration of Competing interests

MPP received travel reimbursement for her work on the new accreditation norms for Accreditation Canada.

## Authors' contributions

MPP carried out the design and coordination of the study. She performed the interviews, the analysis and the first draft. LLC, FC, DA and APC were involved in the study design, gave feedback on the analysis and helped to draft the manuscript. AS was involved in the analysis and helped to draft the manuscript. All authors read and approved the final manuscript.
